# Hormonal Perturbations in Occupationally Exposed Nickel Workers

**DOI:** 10.3889/oamjms.2016.046

**Published:** 2016-03-31

**Authors:** Safia Beshir, Khadiga Salah Ibrahim, Weam Shaheen, Eman M. Shahy

**Affiliations:** *Environmental and Occupational Medicine Department, National Research Centre, Dokki, Giza, Egypt*

**Keywords:** Nickel, FSH, LH, Testosterone, sexual problems

## Abstract

**BACKGROUND:**

Nickel exposure is recognized as an endocrine disruptor because of its adverse effects on reproduction.

**AIM:**

This study was designed to investigate the possible testiculo-hormonal perturbations on workers occupationally exposed to nickel and to assess its effects on human male sexual function.

**METHODS:**

Cross-sectional comparative study, comprising 105 electroplating male non-smoker, non-alcoholic workers exposed to soluble nickel and 60 controls was done. Serum luteinizing hormone, follicle stimulating hormone, testosterone levels and urinary nickel concentrations were determined for the studied groups.

**RESULTS:**

Serum luteinizing hormone, follicle stimulating hormone, urinary nickel and the simultaneous incidence of more than one sexual disorder were significantly higher in the exposed workers compared to controls. The occurrence of various types of sexual disorders (decreased libido, impotence and premature ejaculation) in the exposed workers was 9.5, 5.1 and 4.4 folds respectively than the controls.

**CONCLUSIONS:**

Exposure to nickel produces possible testiculo-hormonal perturbations in those exposed workers.

## Introduction

There is increasing prevalence of various abnormalities in human male reproductive system [1]. It may be due to stress, lifestyle factors and presence of a variety of endocrine-altering environmental chemicals. Occupational activities may involve constant exposure to toxic agents and may have a detrimental effect on human reproduction [1]. As compared to other mammals, human males are of relatively low fertility and hence may be at a greater risk for toxicants influencing reproduction [2].

Nickel is a silver-white metallic chemical element that is naturally present in the Earth’s crust [3]. Pure nickel metal is used in electroplating, as a chemical catalyst, and in the manufacture of alkaline batteries, coins, welding products, magnets, electrical contacts and electrodes. Nickel salts are used in electroplating, ceramics, pigments, and as intermediates (e.g. catalysts, formation of other nickel compounds) [4, 5].

The mammalian male reproductive system can adversely be affected by nickel as shown in many experimental studies [6, 7]. Nickel-induced male reproductive toxic effects coupled with low dietary protein, induce severe changes, including altered physiologic functions, biochemical defects, and structural disorders [6, 7]. Experimental reports suggested that nickel exposure causes decrease in weight of testicular and accessory sex organs: epididymides, seminal vesicles and prostate gland, and decrease in testicular steroidogenic enzymes activities [7-9]. High doses of nickel have shown testicular toxicity involving oxidative stress in mice. This is evidenced by increased lipid peroxidation, DNA damage, and apoptosis in the testes, morphological sperm head abnormalities, and decreased fertility [8, 9].

Relatively few data are available regarding the possible reproductive effects of nickel in human males. Some reports suggested significant positive correlation between high blood nickel level in welders and morphologically abnormal sperms [10, 11]. However, the involvement of oxidative stress mechanisms is the basic mode of action of nickel-induced toxicity in male reproductive dysfunction [12, 13]. Nickel exposure elevates testicular lipid peroxidation and suppresses antioxidant enzyme activities in rats [14]. Kakela and his collegues [6] found that NiCl_2_ induced shrinkage of seminiferous tubules and decreased the number of spermatogonia in the tubules in male rats. Free radical generation from the reaction of Ni-thiol complexes and molecular oxygen, and lipid hydroperoxides could play an important role in the mechanism(s) of Ni toxicity [15].

Nickel was recognized as an endocrine disruptor because of its adverse effect on reproduction [12] and disruption of steroidogenesis and spermatogenesis [16]. It may interfere with the reproductive hypothalamic hormones: lutenizing hormone (LH) and follicle stimulating hormone (FSH) as well as testosterone [17, 18].

The FSH and LH hormones are secreted by the anterior pituitary gland in response to gonadotropin-releasing hormone, produced by the hypothalamus. The secretion of FSH and LH is regulated through negative feedback by steroid hormones and by inhibin (a non-steroidal gonadal substance). In males, LH acts on the Leydig cells in the testes to stimulate the synthesis of testosterone. The action of FSH in maintaining spermatogenesis in the seminiferous tubules of the testes is augmented by LH and testosterone [19]. Alterations caused by endocrine disruptors can be temporary or permanent [20]. Endocrine disruptors can cause reproductive anomalies (morphological and functional gonadal dysfunction, e.g. infertility and decreased libido) and congenital mal-formations (altered embryonic and fetal intrauterine development) [21].

Occupational nickel exposure results in elevated levels of nickel in blood, urine and body tissues^4^. It is known as a potentially harmful element for humans. Its concentration in the environment can rise due to industrial activities [22, 23].

This study was designed to investigate the possible testiculo-hormonal perturbations on workers occupationally exposed to nickel and to assess its effects on human male sexual function.

## Materials and Methods

The study was a cross-sectional comparative study, comprising 105 electroplating male non-smoker, non-alcoholic workers exposed to soluble nickel compound (by inhalation and dermal absorption) in a factory in Helwan district (Cairo, Egypt). Their age ranged from 31 to 60 yr (mean 41.13 ± 5.37) with duration of exposure ranging from 7 to 30 yr (15.41 ± 7.11). The control population included 60 apparently healthy non-smoker, non-alcoholic subjects from the National Research Centre not occupationally exposed to Nickel or other heavy metals, matched for age ranged from 29-59 yr (40.3 ± 6.14).

After taking consent from each individual, they were interviewed and completed a questionnaire that included personal data, detailed occupational history, and marital, sexual, surgical, medical and family histories. Five ml of venous blood sample were collected from all participants either exposed (105) or controls (60) during morning work shift by the authors of the current study. The collected venous blood samples were centrifuged at 3000 rpm for 10 min at 4°C, and the serum was collected and frozen at −20°C for later analysis of serum LH, FSH and testosterone. Urine specimen was collected in plastic containers previously acid washed by 1 M HNO3 and rinsed extensively with water. Also, the urine was frozen and kept at −20°C until analyzed for urinary nickel and urinary creatinine (Cr).

Reproductive endocrine hormones were determined by immunosorbent assay (ELISA techniques) using commercial kits for serum testosterone (Biosource Europe SA Laboratories) and lutenising hormone (LH) (Elitech Diagnostics Laboratories) and follicle stimulating hormone (FSH) (Eurogenetics).

Urinary nickel was determined by the atomic absorption analysis in graphite furnace [24]. The standard addition technique was used for calibration. The nickel content of urine was expressed as micrograms per gram creatinine (μg/g Cr) excretion to compensate for an effect of muscle mass on Cr excretion^25^. Determination of urinary Cr to adjust the values of the urinary nickel was carried out by using the Jaffe method without deproteinsation [26].

### 

#### Data Analysis

The data were statistically analyzed using SPSS18 program. Independent t-test and Chi-square test were used to detect the statistical differences in the quantitative and qualitative data respectively between the two groups. Pearson’s bivariate corre-lation coefficient was also calculated. The differences were considered significant at a level of p < 0.05.

## Results

The urinary nickel and serum testosterone, LH and FSH levels of exposed and control subjects are given in Table 1. There were no significant differences between the control and the exposed groups regarding the age. There was a significant difference in urinary nickel level between the two studied groups. This was accompanied with significantly higher serum concentrations of LH and FSH. At the same time, no difference was found in the levels of serum testosterone.

**Table 1:** The characteristics of the studied groups

Parameters	Control (60) (Mean ± SD)	Exposed (105) (Mean ± SD)	P-value
Age (yr)	40.3 ± 6.14	41.13 ± 5.37	>0.05
Duration of exposure (yr)	-	15.41 ± 7.11	-
Urinary Ni (μg/g Cr)	1.67 ± 0.87	4.18 ±1.50	<0.001
Serum Testosterone(ng/ml)	5.69 ± 1.91	5.44 ± 1.75	>0.05
Serum LH (mIU/ml)	4.47 ± 0.96	6.03 ± 1.23	<0.05
Serum FSH (mIU/ml)	7.19 ± 1.67	8.68 ± 2.47	<0.05

Table 2 shows that 35% of the controls and 42.8% of the exposed group complain of sexual disorders. In the exposed group, there was increase of simultaneous incidence of more than one sexual disorder (33.3%). The distribution of various types of sexual disorders (decreased libido, impotence, premature ejaculation) was higher in the exposed workers. It was found that an odd ratio of occurrence of those sexual disorders among the exposed group was 9.5, 5.1 and 4.4 times the controls respectively (Table 3).

**Table 2:** Incidence of sexual disorders in the studied groups

Parameters	Control (60)		Exposed (105)	
	No	%	No	%
Subjects with sexual disorders	21	35	45	42.8
Subjects complaining of one sexual disorder	15	25	10	9.5
Subjects complaining of more than one sexual disorder	6	10	35	33.3

**Table 3:** Distribution of sexual disorders in the studied groups

Sexual symptoms	Control (60)		Exposed (105)		χ^2^	95% CI		Odds ratio

	No	%	No	%		Lower limit	Upper limit	
Decreased libido	3	5	35	33.3	<0.0001	2.777	32.497	9.5
Impotence	6	10	38	36.2	<0.0002	2.008	12.971	5.1
Premature ejaculation	9	15	46	43.8	<0.0001	1.972	9.898	4.4
Infertility	3	5	8	7.6	>0.05			

Positive correlations were found between the levels of serum FSH and the duration of exposure (r = 0.276, P < 0.05) on one side, and the levels of urinary nickel (r = 0.346, P < 0.01) on the other side.

## Discussion

Nickel can be found practically in all environmental compartments. It originates from natural and artificial sources. The general population is exposed to nickel through nickel alloys and nickel-plated materials, such as coins, steel, and jewelry, and residual nickel may be found in soaps, fats and oils [27]. The urinary nickel level relative to creatinine concentration in normal healthy adults is <2 μg/g [28]. Occupational exposure is common for workers involved in mining, smelting, welding, casting, spray-painting and grinding, electroplating, production and use of nickel catalysts, polishing of nickel-containing alloys, and other jobs where nickel and nickel compounds are produced or used [5]. Nickel was higher in the urine of workers who were exposed to soluble nickel compounds than the workers exposed to less soluble compounds. The levels of nickel in biological fluids increase remarkably in persons with increased occupational or environmental exposure and decline rapidly when exposure is reduced or stopped. Thus, measurements of nickel, particularly in the urine, serum or hair, may serve as indices of exposure [29]. As there is a good correlation between the concentrations of nickel in the air and those present in the urine of exposed subjects, the measurement of urinary nickel represents the most appropriate test for the evaluation of an occupational exposure to this metal [30].

In our study a significant urine nickel levels of the exposed workers compared to the control group may be due to high levels of nickel fumes and fine particles in their work atmosphere. This result was in agreement with results of De Sio et al. [31] and Sancini et al. [32] studies.

A significant high concentration of LH and FSH with no difference in the levels of testosterone was found in the nickel exposed workers in our study, when compared to the control group. Elevated LH and FSH concentrations are sensitive indicators of Leyding and Sertoli cell failure. In many clinically apparent hypogonadism syndromes, LH and FSH are almost invariably raised even when testostrone concentrations are normal [33]. This finding could be explained by direct toxic action of nickel on the testes leading to reduced production of testosterone or by decreasing testicular sensitivity to gonadotrophic actions. In men, intact hypothalamo-pituitary axis is capable of compensating testosterone reduction by increasing FSH and LH secretions, bring back the testosterone production to the normal level.

The nickel interference with the reproductive hypothalamic hormones LH and FSH and the testosterone has been observed in vitro studies and studies on laboratory animals [34, 35]. These results were partly confirmed also in studies on human subjects exposed to nickel [17, 19].

In our study, positive correlations were found between the levels of serum FSH and the duration of exposure and the levels of urinary nickel. These findings were in agreement with that of De Sio et al. [31] who found a positive constant correlation between the values of urinary nickel and plasma FSH. The relationship between urinary nickel and the increase in plasma FSH depends on the action of this metal. The endocrine disruption could be linked to the suppression of the neuroendocrine control in the testicles (with effects on the synthesis and release of testosterone), central nervous system (with effects on FSH, and LH), or both locations simultaneously [36, 37].

The nickel - exposed group in the present study showed significant increase in the incidence of sexual problems in the form of premature ejaculation, impotence and decreased libido, which are the most common male sexual problems as stated by Cleveland Clinic [38].

Testosterone is necessary to maintain male secondary sex characteristics, libido, and probably potency. Thus patients with endocrine abnormalities may present with variety of symptoms, elevated levels of the gonadotropins, FSH and LH in the presence of decreased testosterone levels indicating primary testicular dysfunction.

Future studies are warranted to overcome the current study’s limitation of relatively small sample size.

As a conclusion to the present study, occupational exposure to nickel produces possible testiculo-hormonal perturbations in those workers. Most of the nickel occupationally exposed workers showed compensated primary hypogonadism (elevated LH and FSH values concurrent with normal testosterone concentration) that may be reversible if the exposure ceases.

Medical examination and measurement of urinary level of nickel are recommended periodically for workers occupationally exposed to nickel to detect any side effects or complications of exposure early. Those at high risk should be avoided from further exposure.
